# Characterization of an aerated submerged hollow fiber ultrafiltration device for efficient microalgae harvesting

**DOI:** 10.1002/elsc.202100052

**Published:** 2021-09-12

**Authors:** Franziska Ortiz Tena, Karolína Ranglová, David Kubač, Christian Steinweg, Claudia Thomson, Jiří Masojidek, Clemens Posten

**Affiliations:** ^1^ Institute of Process Engineering in Life Sciences Karlsruhe Institute of Technology (KIT) Karlsruhe Germany; ^2^ Laboratory of Algal Biotechnology Centre Algatech Czech Academy of Science Institute of Microbiology Třeboň Czech Republic; ^3^ iSeaMC GmbH Bremen Germany; ^4^ Faculty of Science University of South Bohemia České Budějovice Czech Republic

**Keywords:** energy, filtration, harvesting, membrane, microalgae

## Abstract

The present work characterizes a submerged aerated hollow fiber polyvinylidene fluorid (PVDF) membrane (0.03 μm) device (*Harvester*) designed for the ultrafiltration (UF) of microalgae suspensions. Commercial baker's yeast served as model suspension to investigate the influence of the aeration rate of the hollow fibers on the critical flux (CF, *J*_c_) for different cell concentrations. An optimal aeration rate of 1.25 vvm was determined. Moreover, the CF was evaluated using two different *Chlorella* cultures (axenic and non‐axenic) of various biomass densities (0.8–17.5 g DW/L). Comparably high CFs of 15.57 and 10.08 L/m/^2^/h were measured for microalgae concentrations of 4.8 and 10.0 g DW/L, respectively, applying very strict CF criteria. Furthermore, the *J*_c_‐values correlated (negative) linearly with the biomass concentration (0.8–10.0 g DW/L). Concentration factors between 2.8 and 12.4 and volumetric reduction factors varying from 3.5 to 11.5 could be achieved in short‐term filtration, whereat a stable filtration handling biomass concentrations up to 40.0 g DW/L was feasible. Measures for fouling control (aeration of membrane fibers, periodic backflushing) have thus been proven to be successful. Estimations on energy consumption revealed very low energy demand of 17.97 kJ/m^3^ treated microalgae feed suspension (4.99 × 10^−3^ kWh/m^3^) and 37.83 kJ/kg treated biomass (1.05 × 10^−2^ kWh/kg), respectively, for an up‐concentration from 2 to 40 g DW/L of a microalgae suspension.

## 1 INTRODUCTION

The term *microalgae* usually refers to photosynthetic microorganisms, both prokaryotic and eukaryotic, forming single cells, filaments, or aggregates. A great variety of species has been discovered to date, revealing various biochemical compounds and possible applications [1]. The fields of food and feed application, wastewater treatment, biofuel, or fertilizer production are just some examples for possible microalgae utilization [2]. As a consequence, dewatering of microalgae is gaining more and more interest as an important part of downstream processing. Cultivating microalgae biomass in outdoor units usually results in dilute suspensions with low biomass densities (measured as dry weight [DW]) of about 1–3 g DW/L (assuming a water content of 90% in the cells this means 10 g/L “solids” correspond to 1–3% w/w total solids [TS]). These values are more than 10 times lower than those achieved in classic heterotrophic cultivation processes. The separation of water from biomass, especially of small single‐celled microalgae strains, thus requires costly processing of large water volumes, representing one of the major challenges of microalgae downstream processing [3, 4, 5].

The majority of microalgae cells are characterized by their small size (range of 1–10 μm [6]) and cell density similar to water (marine algae: 1030–1100 kg/m^3^, freshwater algae: 1040–1140 kg/m^3^ [7]), both resulting in slow settling velocities according to Stokes’ law [8, 9, 10]. In some cases, high lipid content in the cells and high salt content in the medium can even reduce density difference to zero. These cell properties make especially centrifugation rather inefficient. However, even though it is cost and energy demanding, centrifugation is the most commonly used method for harvesting large volumes [4, 5]. It is suitable for most types of microalgae—except fragile species—but mainly applied when high‐value products are required. Disk stack centrifuges (like for yeasts) offering very small sedimentation paths are the device of choice, furthermore special designs have been developed for microalgae harvesting (see a recent review [11]). Filtration—as the major alternative—has its drawbacks as well. The small cell size makes an even thin filter cake in dead‐end filtration practically impermeable. Elasticity of the outer layer of microalgal cells can block the gussets between the particles leading to a so‐called compressible filter cake. In such cases, higher transmembrane pressure (TMP) increases the filter cake resistance but not the flow. Frequently occurring suspended macromolecules make employment of alternative approaches with active filter cake or clogging removal necessary. These could be crossflow filtration, for example, in the construction form of dynamic crossflow filtration [4, 12]. The use of filter aids like in yeast filtration is not applicable. At the end, the produced slurries may contain still too much water for subsequent processing steps. Preconcentration steps like floatation or flocculation (per flocculants or auto‐flocculation per pH‐shifts) are only applicable in medium scale. For the purpose of feed and food additives, wastewater treatment, pharmaceuticals, and bioactive compound production, the application of contaminating substances (like coagulants) that ease the harvesting process is not allowed [3, 4, 9].

**PRACTICAL APPLICATION**

A ready built filtration unit is presented to be employed for large‐scale microalgae cultivation. It is especially foreseen for low energy cell recycle and biomass preconcentration. This saves 90% of water throughput in a subsequent centrifugation step. The presented *Harvester* offers a robust, practical, and low energy suspension toward an energetically feasible microalgae production.

Due to these obstacles, dewatering of microalgae can be technology, energy and cost demanding [10] and can make 20–30% of the biomass production costs [3]. At present, microalgae processing requires a high net energy ratio (energy required to produce dry biomass [DBM] vs. energy content) and carbon balance reducing the application of microalgae biomass mostly to high‐value products (>$10.000 t^−1^) [9]. To be able to set up an economically viable and environmentally sustainable microalgae process, a low‐energy harvesting method is therefore required. Most important, microalgae dewatering processes should be highly effective for most of all microalgae strains generating high biomass concentrations at its recovery. Besides, operation, energy and maintenance costs need to be moderate while handling of large volumes is possible.

Up to now, no universal harvesting technique has been found that meets all requirements. To decrease harvesting costs, dewatering processes are often set up as two step concentration procedures: first step—preconcentration (thickening) and the second step—dewatering. Often, a typical microalgae harvesting process combines membrane filtration followed by centrifugation. Usually, the microalgae slurry is thickened during the first step to 2–7% total suspended solids (TSS) before it is dewatered to a “cake” (paste) of 25% TSS (concentration factor up to 10) [3, 8]. This procedure combines membrane filtration as a low energy step for high water throughput and a high energy centrifugation step to achieve high product concentrations. Preconcentration of, for example , a factor 10 from 3 to 30 g/L and a subsequent centrifugation step from 30 to 300 g/L reduces energy demand in the centrifuge by about 90% compared to centrifugation alone.

Especially in wastewater treatment by microalgae additional tasks have to be accomplished. Low light conditions can lead to low growth rates where the cells cannot take up all nutrients. On the other hand, low biomass concentrations caused by low nutrient availability lead to inefficient light usage. Together with the typical not controllable continuous flow, this requires a controllable biomass recycle or retention. If possible, this should be done with the same filtration device as the preconcentration step. Membrane filtration is—especially for the first concentration step—a dewatering method for microalgae biomass that has several advantages. It is suitable for diluted suspensions with initial concentrations ≤10 wt./vol.% and can yield up to 40% TSS with a microalgae removal of more than 95%. Furthermore, cell damage is minimal due to reduced shear stress, making this generally low‐energy technology also ideal for shear sensitive species. Nevertheless, membrane fouling is the major drawback of this rather slow microalgae harvesting method. Periodic membrane cleaning and/or replacement can increase process costs and reduce the overall process efficiency [3, 8, 9]. It was shown, that energy demand of the two‐step process (first step: membrane filtration, second step: centrifugation) can be effectively reduced by up to 90.4% per m^3^ and 96.9% per kg harvested biomass, respectively [13].

In this work, a prototype of a low‐energy, submerged, aerated hollow fiber membrane filtration unit designed for microalgae harvesting was developed and characterized. The so‐called *Harvester* has been designed to process microalgae cultures of low biomass densities, which should be effectively concentrated. Membrane fouling is minimized by periodic backflushing and due to air bubbling inducing moderate shear on the membrane surface to minimize a cake build‐up and pore blocking. Several variables were evaluated in this study to characterize the membrane performance: membrane permeability and compressibility, critical fluxes (CFs) for various biomass concentrations of unicellular microalgae *Chlorella*. A model organism (yeast *Saccharomyces cerevisiae*) served as control to differentiate between biological and procedural effects.

The *Harvester* described in this study can be regarded as a way to solve the problem of high energy costs of microalgae dewatering during biomass downstream processing.

## 2 MATERIALS AND METHODS

### 2.1 Microalgae cultivation and biomass preparation

#### 2.1.1 Microalgae

Two different *Chlorella* microalgae strains (both cultivated phototrophically) with a cell size between 2 and 10 μm were used for the determination of the CF (see Section 2.3).

*Chlorella vulgaris* H14 (further abbreviated as *Chlorella* A) was cultivated axenically in TAP‐Medium (acetate‐free, pH 7.5) in a closed 28 L photobioreactor (pH 7, 5, 25°C, 1.1 vvm, 1% CO_2_) with internal lightening and light intensities up to 500 μmol/m^2^/h.

The microalgae *C. vulgaris* R‐117 (CCALA 1107, Culture Collection of Autotrophic Organisms, Institute of Botany, Třeboň, Czech Republic; further abbreviated as *Chlorella* B) was cultivated non‐axenically in inorganic medium [14, 15, 16, 17] during July 2020 at Centre Algatech, Třeboň (GPS coordinates – 48°59′15″ N; 14°46′40.630″ E) using an outdoor thin‐layer cascade (650 L). Automatic regulation of CO_2_ supply kept pH at 8.0 ± 0.2.

#### 2.1.2 Yeast

Commercial baker's yeast *S. cerevisiae* (DHW, Vital Gold) was used as a model organism of a spherical cell shape with diameter of 5–10 μm similar to most microalgae species. The yeast experiments allowed defining a preliminary range of operation for the characterization of the filtration device using a microalgae biomass.

The yeast material was dissolved in phosphate‐buffered saline medium (PBS, NaCl 8 g/L, KCl 0.2 g/L, KH_2_PO_4_ 1.44 g/L, Na_2_HPO_4_ 0.24 g/L, pH 7.4), a non‐toxic buffer for cells that protects the cells from osmotic pressure. For CF experiments, two yeast suspensions of different biomass densities of 3.0 and 15.0 g DW/L were used.

### 2.2 Biomass quantification

Biomass density was determined by measurement of the optical density (OD) of microalgae and yeast at 750 and 500 nm, respectively, using a VIS‐spectrophotometer (V‐1200, VWR/Perkin Elmer, Lambda 35).

The measurement of DBM concentration (in g DW per L) was performed as previously described [17, 18, 19]. Culture samples (5 mL) were collected on preweighed glass microfiber filters (GC‐50). The cells were washed twice with deionized (DI) water, the filters were dried in an oven at 105°C for 8 h, and finally transferred to a desiccator and weighed (precision of ±0.01 mg).

### 2.3 Filtration device *Harvester*

#### 2.3.1 Description of the ultrafiltration device *Harvester*

**FIGURE 1:**
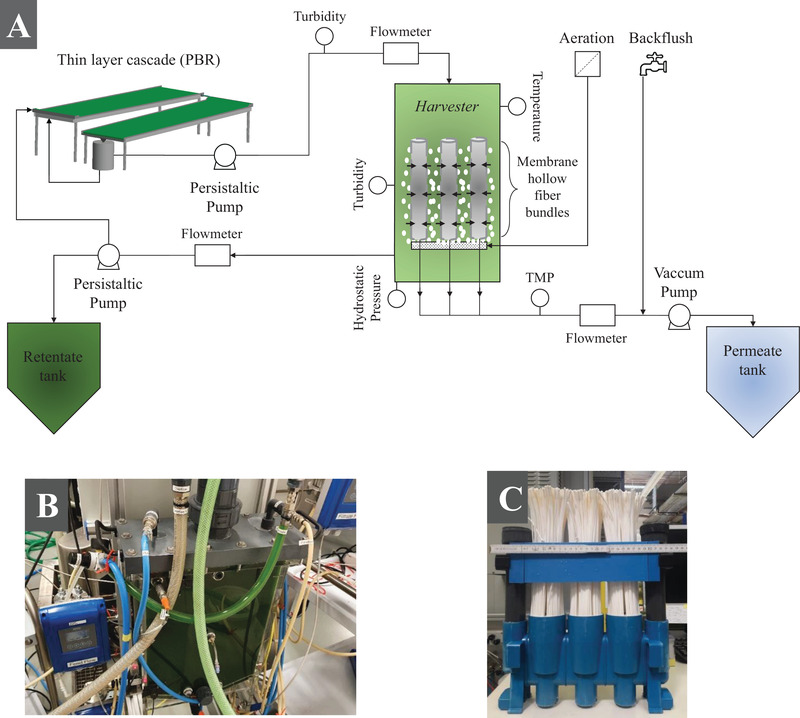
(A) Experimental set‐up of the ultrafiltration device *Harvester*, (B) process of microalgae filtration, (C) membrane module Puron® Hollow Fiber Rows produced by Koch Membrane Systems

For microalgae harvesting, a pilot‐scale ultrafiltration (UF) device (*Harvester 1.0*, designated as *Harvester*) was designed and constructed (Figure 1). The commercially available membrane module (Puron Hollow Fiber Rows, Koch Membrane Systems) used in the *Harvester* consists of three bundles of aerated submerged polyvinylidene fluoride (PVDF) hollow fibers with a nominal pore size of 0.03 μm, the total membrane surface of 1.31 m^2^ and a pure water permeability of about 490 L/m^2^/h·bar at 22°C. The permeate is collected on the inner side of the fibers (outside‐in application). The fibers can be aerated using a controllable mass flow controller (Type 1579, mks) for fouling reduction. The driving force for the filtration process is a TMP, which is applied by a vacuum pump (Drive: MCP‐Z Process ISM918A, Pump head: Z‐201, MI0023, Ismatec) and monitored online using a pressure transmitter (MS – 10663, WIKA). Both constant flux and constant pressure are feasible to generate a permeate flux. A turbidity sensor (Turbimax CUS50D, Endress & Hauser) was used to measure the concentration of the cell‐containing suspension online, fed to the *Harvester* using a peristaltic pump (Flowmaster FMT300 ‐ ISM 1020, Ismatec) from a coupled photobioreactor (PBR in case of microalgae) or a feed tank (yeast). The concentrated cell suspension—retentate—can be pumped out of the *Harvester* via a peristaltic pump (Drive: Ecoline VC‐Easy‐Load ‐ ISM 1077A, pump head: Masterflex L/S ‐ 7518‐10, Ismatec). Cell recycling to the cultivation unit as well as collection in a retentate tank is possible. All fluxes applied (feed, retentate, and permeate) were quantified online using flowmeters (Optiflux 5000, Krohne). The temperature (AT 001, autosen GmbH) and turbidity (Turbimax CUS50D, Endress & Hauser) inside the *Harvester* as well as its filling height (via hydrostatic pressure sensor (AC 004 Niveau, autosen GmbH)) were measured online. Periodic backflushing with tap water was applied to reduce membrane fouling.

#### 2.3.2 Membrane resistance

Prior to the filtration experiments, the membrane was conditioned filtering DI‐water at a constant permeate flux for 45 min. The water‐flux (*J*_W_ in L/m^2^/h) of the clean membrane was measured afterwards for a minimum of 10 min. The membrane resistance *R*_m_ of the clean membrane could be calculated using the applied TMP (in mbar) and temperature‐dependent viscosity of water (*μ*(*T*)) in Pa s according to Equation (1) [20].

(1) $$R_{m}=\frac{\mathrm{TMP}}{J_{W}\;\cdot \mu \left(T\right)}$$

#### 2.3.3 Determination of the critical flux

The critical Flux *J*_c_ was described by Field et al. in 1995 [21] who stated a threshold flux—the so‐called CF—below which fouling does not occur. To date, more classifications (e.g., strong/weak form of the CF) have been defined [22], but those will not be distinguished in this work. The term “critical flux” here refers to the maximum permeate flux, above which a measurable increase in pressure (dTMP/d*t*) occurs at a constant pressure filtration.

Several methods for the measurement of the CF have been used [23]. In the present study, the method described by Diez et al. [24] was applied. The “modified flux‐step method” uses backflushing to remove fouling built‐up during the individual flux steps. In each step, the constant flux was set up for a period of 10 min, within which the TMP was recorded. For evaluation of each interval, the pressure increase (dTMP/d*t*) of each flux step was determined via linear regression. A critical TMP‐increase of 10 Pa/min was chosen analogously to van der Marel et al. [25].

The starting flux and flux‐step height for the various species tested are listed in Table 1. Only the ascending phase was taken into account, as both ascending and descending phases have been proven to identify the same value for the CF (data not shown).

**TABLE 1:** Starting fluxes and flux‐step heights for the CF experiments of various species and aeration rates

Microorganism	Starting flux *J*_Start_	Flux‐step height	
*S. cerevisiae*	9.16 L/m^2^/h 13.75 L/m^2^/h	2.30 L/m^2^/h 2.30 L/m^2^/h	(Aeration 0.00 and 1.25 vvm) (Aeration 2.50 vvm)
*Chlorella* A	10.30 L/m^2^/h 13.70 L/m^2^/h	1.16 L/m^2^/h 2.30 L/m^2^/h	(DBM 1.0 g DW/L) (DBM 0.8 g DW/L)
*Chlorella* B	9.16 L/m^2^/h	0.90 L/m^2^/h	(All biomass concentrations tested)

#### 2.3.4 Evaluation of the filtration process

To evaluate the harvesting efficiency of the complete process, a volumetric reduction factor (VRF) as well as a concentration factor *F*_C_ were defined according to Equations (3) and (4) [26], using the initial (*V*_0_) and final (*V*_f_) volumes as well as the final (*C*_f_) and initial (*C*_0_) microalgae concentrations.

(2) $$\mathrm{VRF}=\frac{V_{0}\;}{V_{f}\;}$$

(3) $$F_{C}=\frac{C_{f}\;}{C_{0}\;}$$

The harvesting efficiency (*η*) was used to evaluate the quality of the permeate generated by the membrane. It refers to the decrease of the OD of the feed suspension (OD_feed_) due to biomass present the permeate (OD_permeate_) in percent. A value of 100% means a full retention of any solid particles by the membrane.

(4) $$\eta \;=\frac{OD_{\mathrm{feed}}\;-\;OD_{\mathrm{permeate}}}{OD_{\mathrm{feed}}}$$

#### 2.3.5 Mass balance for the *Harvester*

The filtration device *Harvester* can be used as a tool for the up‐concentration of a microalgae culture. Furthermore, it can be coupled to a photobioreactor to control the biomass concentration by cell recycling (*R*) or discharge (*D*). A mass balance for the *Harvester* revealed the following relation (Equation (5):

(5) $$c_{x,\mathrm{retentate}}=\frac{{\dot{V}}_{\mathrm{feed}}}{{\dot{V}}_{\mathrm{retentate}}}\cdot c_{x,\mathrm{feed}}$$

where ${\dot{V}}_{\mathrm{feed}}$ is the feed flux into the *Harvester* in L/min, $c_{x,\mathrm{feed}}$ is the biomass concentration (g DW/L) of the feed suspension, ${\dot{V}}_{\mathrm{retentate}}$ the retentate flux out the *Harvester*, and $c_{x,\mathrm{retentate}}$ the biomass concentration (g DW/L) of the retentate.

#### 2.3.6 Energy consumption

To evaluate the energy consumption of the filtration unit *Harvester*, the pumping of the feed suspension (feed pump), permeate (permeate pump) as well as the concentrated retentate stream (harvest pump), together with the energy needed for membrane aeration have to be considered. For various biomass concentrations (start/end), the VRFs, and concentration factors, the energy demand for different scenarios could be evaluated and compared. A theoretical set‐up with a given feed flux of 100 m^3^/h, an aeration rate of the membrane fibers of 1.25 vvm, and an operational permeate flux set to a sub‐critical value of 19.5 L/m^2^/h (representing 85% of the CF predetermined for representative biomass concentrations) were considered. Using the above‐mentioned frame conditions, the energy required to perform a biomass concentration to a certain level per m^3^ permeate (*E*_v_ in kJ/m^3^ and kWh/m^3^) was calculated. Furthermore, the energy consumption per kg DW of the harvested biomass *E*_w_ (in kJ/kg and kWh/kg) could be determined.

The theoretical pumping power requirement (*P*_th,feed/retentate_) of the peristaltic feed and harvest pump was calculated using Equation (6) with$\;\dot{V}$ is the corresponding flux (feed/retentate), ρ is the density of the suspension pumped (feed/retentate), g the gravity acceleration, and H is the pumping height.

(6) $$P_{\mathrm{th},\mathrm{feed}/\mathrm{retentate}}=\dot{V}\cdot \rho \cdot g\cdot H$$

For the permeate pump, *P*_th,permeate_ was determined using Equation (7), $\dot{V}$ being the permeate flux and Δ*p* the pressure difference between both sides of the membrane counted positive from outside to inside of the fibers.

(7) $$P_{\mathrm{th},\mathrm{permeate}}=\dot{V}\cdot \Delta p$$

Based on the results in Section 3.2 (characterization of the *Harvester*), the TMP needed to generate a permeate flux of 19.5 L/m^2^/h is set to 136 mbar, considering fouling effects. Furthermore, the mean hydrostatic pressure above the membrane is taken into account. This value is thus taken as a minimum threshold value to generate a permeate flux in this range.

The actual power demand of all three pumps *P*_S_ was calculated by dividing the theoretical demand *P*_th_ by the pump‐specific efficiency factor *η* (Equation (8)):

(8) $$P_{S}=\frac{P_{\mathrm{th}}\;}{\eta \;}$$

The energy required for the aeration of the membrane fibers *P*_a_ was calculated using Equation (9), including the aeration rate $\left({\dot{V}}_{\mathrm{air}}\right)$ $\left({\dot{V}}_{air}\right)$ in m^3^/s together with the hydrostatic pressure above the gas outlet ($p_{\mathrm{hydro}}$) due to the water column.

(9) $$P_{a}={\dot{V}}_{\mathrm{air}}\cdot p_{\mathrm{hydro}}$$

## 3 RESULTS AND DISCUSSION

### 3.1 Membrane characterization

#### 3.1.1 Critical flux experiments using baker's yeast

Prior to the start of each filtration experiment, DI‐water was filtered at 23 L/m^2^/h for 45 min. The filtration data was used to calculate the membrane resistance *R*_m_ for every approach according to Equation (1) (see Supporting Information Figure S1).

Five sets of experimental conditions regarding DBM concentration and aeration rates of the membrane fibers were applied to determine the *J*_c_ value for yeast suspensions (see Table 2). In line with other studies [23, 27–32], a decrease of the CF (from 25.18 to 20.60 L/m^2^/h) with increasing biomass concentrations (from 3.0 to 15.0 g DW/L) for an aeration rate of 1.25 vvm was measurable. Yet, the effect of cell concentration on CFs has not been fully clarified—changes in viscosity or the diffusion coefficient and surface interactions are described theories [23].

**TABLE 2:** *J*_c_ and CF criterion dTMP/d*t* of a yeast suspension (*S. cerevisiae*) for various biomass concentrations (3.0 and 15.0 g DW/L) and various aeration rates of membrane fibers of 0.00, 1.25, and 2.50 vvm

Biomass concentration (g DW/L)	Aeration (vvm)	*J*_c_ (L/m^2^/h)	dTMP/d*t* at *J*_c_ (Pa/min)
3.0	0.00	*J*_c_ > 27.48	4.32
3.0	1.25	25.18	11.01
15.0	1.25	20.60	20.61
3.0	2.50	*J*_c_ > 27.48	4.04
15.0	2.50	18.32	22.77

Aeration of the membrane surface can have two opposite effects: on the one hand, air bubbles create shear forces along the membrane surface, causing the transportation of particles into the bulk phase and thereby a reduction of the build‐up of a filter cake as well as concentration polarization [32, 33, 34, 35], which is the case for low cell concentrations (3.0 g DW/L) in this study.

On the other hand, vigorous bubbling increases cell stress and can even lead to its rupture [34, 36–38]. Cell debris together with exposed small intracellular substances has been identified as main reasons for membrane fouling [34, 35, 38–40]. For a biomass concentration of 15.0 g DW/L, a slight decrease of *J*_c_ was observed, when aeration was doubled. Foam formation was present during this experiment indicating protein release due to cell rupture caused by high shear rates created by the augmented bubbling.

The effect of aeration intensity on the CF using a submerged flat sheet membrane module was studied [28]. The authors found a linear increase of the CF with augmenting aeration rate for cell concentrations (sludge) between 9.6 and 22.6 g DW/L. The slope of the curves and thereby the influence of the aeration rate became more significant for high cell densities. The data also indicated a slight reduction of *J*_c_ when exceeding a specific bubbling rate (about 1.0 vvm), which is in line with the presented results. Alipourzadeh et al. investigated the effects of biomass concentration (*C. vulgaris*) and aeration rate of the membrane surface on the filtration performance of a submerged flat sheet membrane [37]. Their model‐supported studies showed an optimal aeration rate of 1.25 vvm to reduce fouling effects on the membrane surface (mainly cake build‐up), which is consistent with the results of this work. The positive impact of bubbling was proven to be more significant at lower biomass concentrations (∼0.65 g DW/L) implying a prevalent effect of the increased fouling caused by cells compared to the reductive effect of the air bubbling. In general, aeration enhances the turbulence along the membrane and helps to reduce the accumulation of microalgae cells. Bubbling intensities above the optimal value can cause the development of shear forces resulting in cell rupture and even amplified irreversible fouling effects [37].

Without aeration, the CF for the yeast suspension (3.0 g DW/L) was not reached within the flux interval tested, indicating a high CF of more than 27.48 L/m^2^/h. This was not expected as cells can easily form a cake layer on the membrane surface without bubbling. During the experiment, sedimentation inside the filtration chamber of the *Harvester* was visible, accompanied by an obvious dilution and a slight color change of the yeast culture. *S. cerevisiae* is a facultative anaerobic microorganism; it can switch its metabolic activities to anaerobic fermentation when leaking oxygen, which can occur in the absence aeration of the membrane fibers. However, no energy source (sugar) was provided in the buffer suspension to generate the yeast suspension. The absence of sugar and oxygen to maintain the basic cellular metabolism can explain the described differences of this experimental set‐up. Hence, the changes of the biomass as well as the cell concentration during the experiment did not allow to evaluate and compare the CF correctly under the given conditions. To conclude, the physiological performance of the biomass is important for a filtration process as well as its evaluation.

#### 3.1.2 Critical flux experiments with microalgae

Prior to each filtration experiment, DI‐water was filtered at 20–23 L/m^2^/h for 45 min. The filtration data were used to calculate the membrane resistance *R*_m_ for every approach according to Equation (1) (see Supporting Information Figure S1).

**FIGURE 2:**
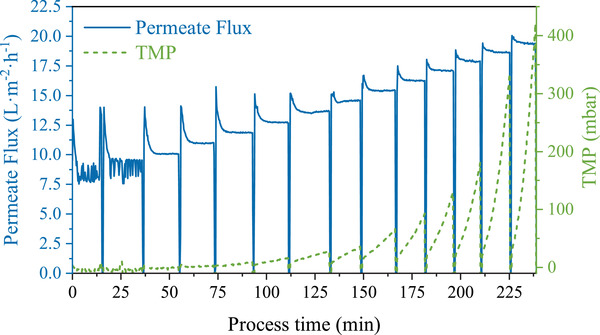
Exemplary permeate‐flux and TMP time profiles for the flux stepping method using *Chlorella* B (10.0 g DW/L) at an aeration rate of 1.25 vvm

Figure 2 demonstrates a typical permeate‐flux and TMP time profile (*Chlorella* B, 10.0 g DW/L, 1.25 vvm) for the CF experiments conducted applying the flux‐step method. An increase in TMP of >10 Pa/min at a constant permeate flux was used as CF criteria.

**FIGURE 3:**
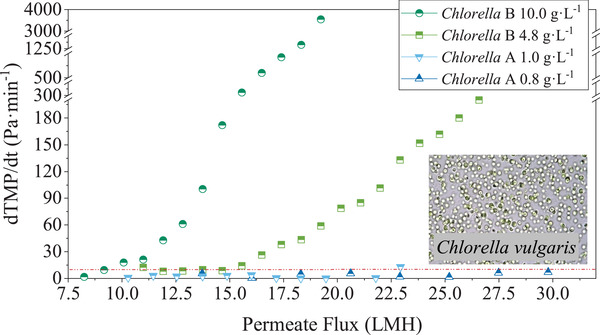
Dependence of dTMP/d*t* versus permeate flux of *Chlorella* cultures (*Chlorella* A – axenic, *Chlorella* B – non‐axenic) at various biomass concentrations (0.8, 1.0, 4.8, 10.0, and 17.5 g DW/L) under an aeration rate of 1.25 vvm

Microalgae biomass grown in any medium contains—apart from cells—cell debris as well as small soluble molecules (extracellular organic matter, EOM) produced by the microalgae metabolism. Several studies have revealed the fouling propensity of all constituents of such suspensions [20, 26, 40]. EOM has been identified as to cause irreversible fouling, resulting in a permanent blockage of the membrane pores. Cells alone are responsible for the build‐up of reversible filter cakes on the membrane surface that can be nearly totally removed by backflushing. The consortium of cells, cell debris and EOM can create dense filter cakes on the membrane surface that increase the filtration resistance but help to reduce irreversible fouling effects [34, 35, 39]. Compared to the model culture (yeast) in the previous section, the filtration of *Chlorella* cultures resulted in higher rates of pressure‐increase (dTMP/d*t*) and lower CFs due to the presence of EOM and cell debris (see Tables 2 and 3).

**TABLE 3:** *J*_c_ and CF criterion dTMP/d*t* of *Chlorella* cultures (*Chlorella* A – axenic, *Chlorella* B – non‐axenic) at various biomass concentrations (0.8, 1.0, 4.8, 10.0, and 17.5 g DW/L) with an aeration rate applied on the membrane fibers of 1.25 vvm

Microalgae species	Biomass concentration (g DW/L)	Aeration (vvm)	*J*_c_ (L/m^2^/h)	dTMP/d*t* at *J*_c_ (Pa/min)
*Chlorella* A	0.8	1.25	*J*_c_ > 32.06	6.95
*Chlorella* A	1.0	1.25	*J*_c_ > 22.93	12.11
*Chlorella* B	4.8	1.25	15.57	14.45
*Chlorella* B	10.0	1.25	10.08	18.04
*Chlorella* B	17.5	1.25	10.08	15.27

Consistent with the results in Section 3.1.1, a decrease of the CF was measured with increasing cell concentration. In all cases, a negative linear correlation of DBM (of 1.0–10.0 g DW/L) and *J*_c_ was found (*R* ^2^ = 0.97), which is in line with other studies [29, 41]. Contrary to those findings, *J*_c_ stagnated for higher cell concentrations (10.0 and 17.5 g DW/L), which was accompanied by a lower pressure increase (dTMP/d*t*, Table 3) measured for the highest biomass concentration. In contrast to these findings, the time profiles of the permeate flux and TMP showed an obviously higher fouling occurring for the higher concentrated microalgae culture: a stable permeate flux above 15 L/m^2^/h could not be achieved for this culture despite higher setpoints (see Supporting Information Figure S2). A flux decrease could be observed, although the TMP is increasing constantly up to 150 mbar. Preliminary experiments revealed that the membrane used is compressible resulting in a pressure‐dependent membrane resistance *R*_m_ (Equation (10), *R* ^2^ = 0.98, see Supporting Information Figure S3):

(10) $$R_{m}=4.135\times 10^{11}+2.004\times 10^{10}\cdot \mathrm{TM}P^{0.6648}$$

In consequence, the membrane pores were “squeezed” together and therefore the membrane resistance was increased, which led to a decrease of the flux at a specific TMP. In this case, the CF was not suitable to depict the differences in fouling behavior between the two suspensions with the biomass concentration of 10.0 and 17.5 g DW/L.

A higher increase of the pressure with time (dTMP/d*t*, see Figure 2) was observed for the denser *Chlorella* B cultures (non‐axenic, 4.8 and 10.0 g DW/L) compared to the low cell concentrations (0.8 and 1.0 g DW/L). This can be attributed to the increased biomass concentration as already discussed. Nevertheless, other factors need to be considered, namely microalgae species, culture variability, and cultivation conditions.

Microalgae species: As indicated by the microscopic pictures, the cell size of both *C. vulgaris* strains was within the range of 2–10 μm, as previously reported [42]. The differences in filtration performance have usually been attributed to cell surface characteristics, which can influence the interaction of cell and membrane surface together with the amount and varieties of EOM produced by the microalgae metabolism. Small molecules like EOM can enhance interactions between solid particles as well as with the membrane enhancing membrane fouling [35, 39, 43]. Without further investigation, no clear conclusion can be drawn about those aspects.
Culture composition: Non‐axenic *Chlorella* B culture was cultivated outdoors in an open reactor system and thus, some bacteria might be present which are absent in the axenic culture (*Chlorella* A). It is well known that the structure and density of filter cakes on membrane surfaces are influenced by the composition of the cultures to be filtered [35, 39]. Small solid particles usually cause high filtration resistances whereas larger particles create high porous filter cakes. Furthermore, the size distribution of solid particles influences the structure of the filter cake occurring: Consortia with large particle size distributions tend to increase the packing density of the building‐up filter cake structure and thereby its additional resistance to filtration. Voids between larger particles within the cake are filled by smaller particles resulting in a high cake density [44, 45]. The cell size distribution in the *Chlorella *B culture was wider compared to the *Chlorella *A culture due to the presence of bacteria, which are usually smaller than microalgae cells [1]. Therefore, an influence of the cell size distribution to the increase of the TMP (dTMP/d*t*) cannot be neglected.
Culture conditions: The culture conditions of the two *Chlorella* cultures used were different. The culture of *Chlorella *A originates from a large‐scale laboratory reactor with controlled conditions. In contrast, *Chlorella *B was grown outdoors under natural conditions (concerning temperature and light).The microalgae cultures were thus exposed to an unstable and not‐optimized environment. Unfavorable conditions like low temperatures or high irradiance can lead to cell stress accompanied by an increased content of cell debris and/or a higher production of EOM resulting in higher membrane fouling.

To conclude, the results showed a linear increase of the CF with increasing biomass concentration up to 10.0 g DW/L equivalent accompanied by rising membrane fouling. Higher cell concentrations lead to more pronounced fouling but cannot be simply detected by the *J*_c_ due to the necessity of high forces leading to a membrane compressing. Additionally, the variation in the culture composition and conditions can be considered to explain higher dTMP/d*t* rates of *Chlorella *B cultures compared to *Chlorella *A.

An overview of several studies measuring the CF of various microalgae, mostly *Chlorella* species using microfiltration (MF) and UF membranes is shown in Table 4. Various microalgae species have been tested, whereat *Chlorella* occurred the most. The data illustrates several differences and trends concerning membrane pore size, cell size, and biomass concentration influencing the CF.

**TABLE 4:** Comparison of CFs for various microalgae species and biomass concentrations using submerged microfiltration (MF) and ultrafiltration (UF) membranes. The CF criterion is an important factor for the evaluation of *J*_c_, which is mainly influenced by the pore size of the membrane and the biomass concentration of the suspension applied

Membrane	
Configuration	Specification	Microalgae species (cell size in μm)	Biomass concentration (g DW/L)	*J*_c_ (L/m^2^/h)	CF Method CF criterion Starting flux *J*_Start_ (step height)	Ref
Submerged, external, flat sheet	MF PVDF 0.1 μm	*Chlorella pyrenoidosa*	0.3	27 (15°C) 30 (25°C) 42 (35°C)	FS‐IFM 20 Pa/min *J*_Start_ 15 L/m^2^/h (3 L/m^2^/h)	[46]
Submerged, internal, flat sheet	MF PVDF 0.1 μm	*Chlorella pyrenoidosa*	0.3	42	FS‐IFM 20 Pa/min *J*_Start_ 10–15 L/m^2^/h (2.5–3 L/m^2^/h)	[47]
Submerged, internal, hollow fibers	MF HDPE 0.4 μm	*Chlorella* sp. ADE4	1.0	58.5	FS 0.2 kPa/min *J*_Start_ 42 L/m^2^/h (12–33 L/m^2^/h)	[48]
Submerged, external, hollow fibers	UF PVDF 0.03 μm	*Chlorella vulgaris* (2–10 μm)	0.0691 0.3709	39.4 16.6	FS‐IFM‐R 20 Pa/min –	[29]
Submerged, internal, flat sheet	UF PVDF 0.036 μm	*Chlorella vulgaris* (2–10 μm)	0.41	>50	FS‐IFM 10 Pa/min *J*_Start_ 10 L/m^2^/h (5 L/m^2^/h)	[49]
		*Phaeodactylum tricornutum* (8–35 μm)	0.23	>50		
Submerged, internal, flat sheet	UF PVDF 0.03 μm/0.05 μm	*Chlorella pyrenoidosa*	0.3	20/25	FS‐IFM 20 Pa/min *J*_Start_ 10–15 L/m^2^/h (2.5–3 L/m^2^/h)	[47]
Submerged, external, hollow fibers	UF PVDF 0.03 μm	*Chlorella vulgaris* (2–10 μm)	0.8 1.0	>32.06 >22.93	FS‐B 10 Pa/min	This study
		*Chlorella vulgaris* (2‐10 μm)	4.8 10.0 17.5	15.57 10.08 10.08	*C*: *J*_Start_ 10.30–13.7 L/m^2^/h (1.16–2.30 L/m^2^/h) *D*: *J*_Start_ 9.16 L/m^2^/h (0.9 L/m^2^/h)	
Submerged, internal, flat sheet	UF PVDF 0.036 μm	*Chlorella vulgaris* (2–10 μm)	0.21 0.73 1.43	>50 40 35	FS‐IFM 10 Pa/min *J*_Start_ 10 L/m^2^/h (5 L/m^2^/h)	[13]
		*Phaeodactylum tricornutum* (8–35 μm)	0.25 0.79 1.52	>50 45 30		
Submerged, external, flat sheet	UF Cellulose MWCO: 10 kDA	*Chodatella* sp.	2 × 10^6^ cells/mL OD_684_ 0.472	105	TMP‐Step – TMP_Start_ 35 kPa Step: 35 kPa	[50]
		*Chlorella vulgaris* (2–10 μm)	2 × 10^6^ cells/mL OD_689_ 0.652	70		
		*Microcystis* sp.	2 × 10^6^ cells/mL 324 NTU	55		
Submerged, external, flat panel	UF PES‐PVD 0.05 μm	*Isochrysis* (3—5 μm) *Chlorella vulgaris* (2–10 μm)	0.30 0.40	15 50	FS‐B/FS‐R – *J*_Start_ 10 L/m^2^/h (10 L/m^2^/h)	[30]
		*Phaedactylum tricornutum* (8–35 μm)	0.30 5.00 10.00	50 45 40		
		*Pavlova lutheri* (5–7 μm)	0.90	20		
		*N. oculata* (1–3 μm)	1.60 8.86 10.00	35 10–20 15		

Internal: inside PBR; external: separate from PBR; MF: microfiltration; UF: ultrafiltration; PVDF: polyvinylidene fluorid; HDPE: high‐density polyethylene; PES‐PVD: polyethersulfone polyvinylpyrrolidone; MWCO: molecular weight cut‐off; CF: critical flux; FS: flux‐stepping; IFM: improved flux‐step method; ‐B: backflushing; ‐R: relaxation.

CFs for MF membranes are typically higher for the comparable cell concentrations and species as compared to UF membranes. This is valid for example for the culture of *C. pyrenoidosa* at a biomass concentration of 0.3 g DW/L, the CF is higher for the MF membrane compared to the UF membrane [47]. This effect can be explained by the usually lower resistance of MF membranes due to the larger pore size and thereby higher permeability. Furthermore, cell size seems to inversely influence CF values: Microalgae species with smaller cell size, for example , *Isochrysis*, tends to have lower values (15 L/m^2^/h) as compared to species with greater cell size (e.g., *C. vulgaris*, *J*_c_ = 50 L/m^2^/h) at similar biomass concentrations (0.3–0.4 g DW/L), due to higher diffusion activities to the bulk phase and lower surface interactions with the membrane of smaller particles compared to bigger ones [23]. Microalgae cells of similar size (*Nannochloropsis oculata*, *C. vulgaris*) achieve comparable values for *J*_c_ (35 L/m^2^/h) for analogous culture densities (1.43–1.6 g DW/L) [13, 30]. The membrane material seems to have only little influence on the CF as comparable values for similar species and biomass concentrations of different, independent studies have been shown [23].

It must be emphasized that the CF‐criterion in this study was set rather low to 10 Pa/min. This is an important variable for the evaluation of *J*_c_ and needs to be taken into account when comparing those values. Keeping this fact in mind it can be concluded that the filtration performance (as measured by the CF) of the filtration device *Harvester* presented in this study falls within this range, or even prevails comparable set‐ups.

The CF defines the upper limit of the membrane performance, where a stable filtration process without severe fouling can be performed. A filtration device can never be run at its maximum as to avoid capacity overload and to guarantee its optimal efficiency. Flux values either applied for microalgae harvesting (first step of dewatering or up‐concentration) or as a part of a microalgae membrane bioreactor (internal or external) are set below the threshold of the membranes used [39, 51, 52] to sub‐critical values of, for example , 85% of *J*_c_ [13]. Therefore, *Harvester* can be classified as suitable for both criteria addressing microalgae harvesting.

### 3.2 Microalgae filtration tests

In order to characterize the filtration performance of the *Harvester* (“proof‐of‐concept”), the cultures of *Chlorella *B at four biomass densities were prepared and filtered to test the capacity of the membrane. The VRF, concentration factor (*F*_C_), and the harvesting efficiency (*η*) were calculated according to Equations (2)–(4) in Table 5. Due to limited time, all filtration experiments were restricted to a maximum of 1–4 h.

**TABLE 5:** VRF and concentration factor (*F*_C_) for filtration of the *Chlorella* B cultures using the described filtration device *Harvester*

Biomass concentration (g DW/L)				
Start	End	Aeration (vvm)	VRF	*F_C_*
1.53	19.00	1.25	11.5	12.4
2.30	16.20	1.25	7.7	7.0
5.80	24.60	1.25	4.5	4.2
14.20	40.00	1.25	3.5	2.8

**FIGURE 4:**
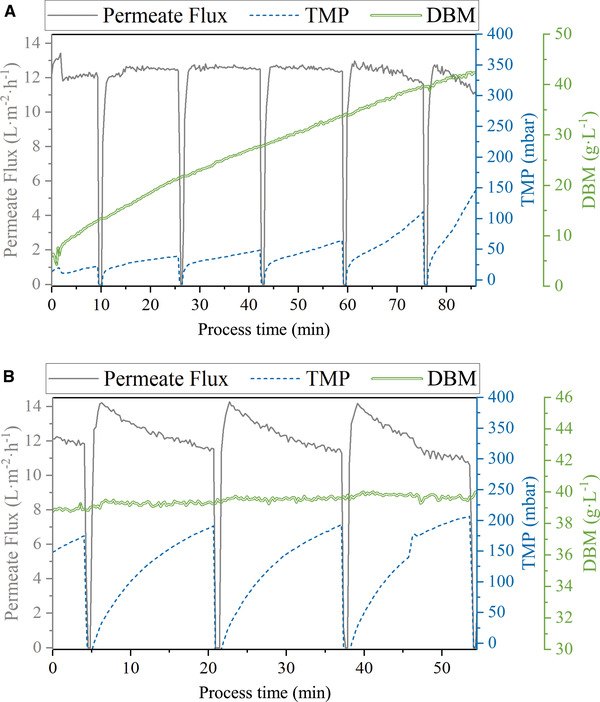
Permeate‐flux, TMP, and DBM time profiles for different modes of operation (A: up‐concentration *c*_Harvester_: 14.2 → 40 g DW/L, B: continuous filtration *c*_Harvester_ = 40.0 g DW/L) for the *Chlorella* B culture

When the culture of biomass density of 1.53 g DW/L was used a maximum *F*_C_ of 12.4 and VRF of 11.5 could be achieved within the short time of testing. Furthermore, for initially denser *Chlorella* cultures, cell densities of up to 40 g DW/L (in retentate) are achievable by the *Harvester*. Fouling control (aeration of the membrane fibers and periodic backflushing) was thus effective and allowed to set up a stable filtration process even for high biomass concentrations (Figure 4). An up‐concentration of the microalgae cells (e.g., from 14.20 to 40.00 g DW/L) as well as a continuous filtration of a biomass flux (14.20 g DW/L) producing a constant retentate stream of 40.00 g DW/L is feasible using the *Harvester*, as shown in Figure 4. The harvesting efficiency *η* varied between 78% and 93% within the first 30 min of each trial but went up to >99% after this short starting period.

According to the mass balance (see Equation (5) in Section 2.3.5), the biomass concentration in the retentate of the filtration device (*c*_X,retentate_) was influenced by the cell concentration in the feed/PBR (*c*_X,feed_) as well as by the quotient of feed and retentate flux (${\dot{V}}_{\mathrm{feed}}$, ${\dot{V}}_{\mathrm{retentate}}$). Turbidity measurements inside the *Harvester* and the PBR proofed the capability of the filtration device: the calculated concentration *c*_X,retentate_ was achieved after a short time (∼20 min) with very low deviations (<1%). Furthermore, an up‐concentration of the biomass of more than factor 2 (from 14.20 to 40.00 g DW/L) was reached in this experiment.

### 3.3 Energy consumption

Table 6 summarizes the operational and device parameters used for the calculation of the energy demand of the *Harvester* for different process scenarios applying an arbitrarily chosen feed inflow of 100 m^3^/h. It needs to be mentioned that *P*_th,feed_ and *P*_th,retentate_ strongly depend on the local circumstances and are given here only as examples, while the energy used to generate the permeate flux is a central element subject of this investigation. The required pump power is calculated considering a pump efficiency factor, which was set exemplary to 0.7. This value usually varies between 0.6 and 0.8, depending on the pump used and is thus not crucial for a general idea of the plant performance. The energy needed for aeration is neglectable compared to the power requirement of the pumps (Table 6). Constructive optimizations need to be performed when applying the principle of the *Harvester* in large‐scale and/or long‐term operation to reduce the power demand of the feed pump, which is easily feasible. The energy required to maintain the TMP (*P*_th,permeate_, Table 6) is thus the main power sink.

**TABLE 6:** Operational and plant parameters used for calculation of energy consumption of the *Harvester*

Membrane aeration rate	Membrane surface	Permeate flux	Transmembrane pressure	Feed flux
1.25 vvm	1.31 m^2^	19.5 L/m^2^/h	13 635 Pa	100 m^3^/h
2.5 × 10^−4^ m^3^/s		7.1 × 10^−6^ m^3^/s		2.8 × 10^−2^ m^3^/s
*P*_th,permeate_ (kJ/s)	*P*_th,feed_ (kJ/s)	*P*_th,retentate_ (kJ/s)	*η* pumps (all)	Power demand aeration (kJ/s)
0.303	0.332	0.023	0.70	1.194 × 10^−3^

Different up‐concentration scenarios are considered resulting in varying values for *F*_C_ and VRF (see Table 7). For this purpose, the biomass concentration in the input *Chlorella* culture (*c*_x,Start_) is specified, as well as the desired concentration in the retentate (*c*_x,End_).

**TABLE 7:** Energy consumption by the filtration device *Harvester*: energy required per m^3^ permeate (*E*_v_) and per kg of DBM in retentate (*E*_w_) for different concentration scenarios

			*E*_v_		*E*_w_	
Concentration proportion*c*_x,Start_ → *c*_x,End_ (g DW/L)	*F*_C_	VRF	kJ/m^3^	kWh/m^3^	kJ/kg	kWh/kg
2 → 10	5	5	42.35	1.18 × 10^−2^	16.94	4.71 × 10^−3^
3 → 30	10	10	39.17	1.09 × 10^−2^	11.75	3.26 × 10^−3^
2 → 40	20	20	37.83	1.05 × 10^−2^	17.97	4.99 × 10^−3^

Compared to other studies [13, 53], a low energy demand was calculated for all scenarios considered (Table 7). Even for a rather high up‐concentration from 2 to 40 g DW/L, the energy demand does not exceed 1.05 × 10^−2^ kWh/m^3^ permeate or 4.99 × 10^−3^ kWh/kg harvested microalgae biomass, respectively. Nevertheless, biomass concentrations suitable for the final process step in algae dewatering (e.g., drying) should reach between 150 and 250 g DW/L [8], which is not feasible using membrane harvesting. Therefore, the estimated energy consumption of a two‐step microalgae harvesting process (first step: membrane filtration using the *Harvester*, second step: centrifugation) is illustrated in Table 8. Three scenarios are compared: (I) one‐step dewatering applying centrifugation alone, (II) two‐step process with fivefold up‐concentration via filtration followed by centrifugation, and (III) two‐step process with 20‐fold up‐concentration via filtration followed by centrifugation. The data required to calculate the energy consumption of the centrifugation was taken from literature [8, 13], assuming an energy demand of 7.99 kWh/m^3^. Table 8 demonstrates the predominance of a coupled dewatering process compared to one‐step centrifugation: An energy reduction of ∼80% per m^3^ can be achieved even for a comparably low up‐concentration of fivefold using the *Harvester*. For a preconcentration of 20‐fold, the energy demand per kg harvested microalgae biomass is reduced by more than 99%. Assuming the cost of 10 cent/kWh (Germany), a microalgae dewatering process is thus economically feasible (max. 16.1 cent/m^3^ and 1.65 cent/kg microalgae, respectively), if using this two‐step process introduced, compared to the one‐step centrifugation. The energy price—of course—can vary from region to region, thus the exact economic feasibility needs calculated case from case individually.

**TABLE 8:** Energy consumption for microalgae harvesting for three scenarios combining filtration and centrifugation (for two preconcentrations factors *F*_C_) or solely centrifugation (*c*_x,Start_: 2 g DW/L, *c*_x,End_: 250 g DW/L). The energy required per m^3^ permeate (*E*_v_) and per kg of DBM in retentate (*E*_w_) are compared for a continuous algae feed of 100 m^3^/h

	Membrane filtration			Centrifugation		
Scenario	*F*_C_	*E*_v_ (kWh/m^3^)	*E*_w_ (kWh/kg)	*F*_C_	*E*_v_ (kWh/m^3^)	*E*_w_ (kWh/kg)	*E*_v_ total (kWh/m^3^)	Red.‐%	*E*_w_ total (kWh/kg)	Red.‐%
I	–	–	–	125	7.99	3.99	7.99	0.00	3.995	0.00
II	5	1.18 × 10^−2^	4.71 × 10^−3^	25	1.60	0.16	1.61	79.85	0.165	95.87
III	20	1.05 × 10^−2^	4.99 × 10^−3^	6.25	0.40	0.01	0.41	94.87	0.015	99.62

Scenario I: direct up‐concentration to desired DBM concentration only using centrifugation, Scenario II: two‐step up‐concentration using the *Harvester* as first step (fivefold concentration) followed by centrifugation as second step, Scenario III: two‐step up‐concentration using the *Harvester* as first step (20‐fold concentration) followed by centrifugation as second step. Red.‐%: relativeenergy reduction of two‐step harvesting using membrane filtration compared to direct up‐concentration only using centrifugation

Summing up, using the *Harvester*, a microalgae suspension can effectively be up‐concentrated as first step in the downstream process of microalgae biomass, which can then be followed by centrifugation to maintain high cell concentrations, as demonstrated in Tables 7 and 8. Membrane filtration can thus reduce the energy demand per kg DBM significantly (up to about 99%, Table 8) when coupling it to centrifugation [30]. Furthermore, the low energy demand allows the *Harvester* to be applied for cell recycling in continuous microalgae cultivation, for example , for the production of low‐cost biomass or wastewater remediation.

## 4 CONCLUSION

The present study revealed the suitability of the submerged aerated PVDF membrane UF device *Harvester* for microalgae harvesting. Comparable high fluxes (10.08 to >32.06 L/m^2^/h) can be realized for different biomass concentrations (0.8–17.5 g DW/L). Optimal operational conditions (fouling control via membrane aeration and backflushing) allow a stable filtration handling high biomass concentrations (up to 40.0 g DW/L) efficiently. The very low energy demand makes the *Harvester* an ideal tool for the first up‐concentration step in microalgae downstream processing. Further, it can be used for external cell recycling in continuous microalgae cultivation, e.g., deployed for wastewater treatment.

## NOMENCLATURE

**Table:** 

Symbol	Units	Explanation
$\dot{V}$	L/min	Volumetric flow rate
*μ*(*T*)	Pa s	Temperature‐dependent viscosity
‐B		Backflushing
*C*	g/L	Concentration (Indices: 0: Start, f: final, x: biomass)
*C. vulgaris*		*Chlorella vulgaris*
CF	Critical flux	
DW		Dry weight
*E*	kJ/kWh	Energy (indices: v: per m^3^ permeate, w: per kg algae biomass)
EOM		Extracellular organic matter
F_C_	–	Concentration factor
FS		Flux‐stepping
*g*	kg m/s^2^	Gravity acceleration
*H*	m	Pumping height
HDPE		High‐density polyethylene
IFM		Improved flux‐step method
*J*	L/m^2^/h	Permeate flux
*J*_c_	L/m^2^/h	Critical flux
MF		Microfiltration
MWCO		molecular weight cut‐off
OD		Optical density
*P*	–	Power (indices: th: theoretical, s: pump specific, a: aeration)
*p*	bar	Pressure (index hydro: hydrostatic)
PES‐PVD		Polyethersulfone polyvinylpyrrolidone
PVDF		Polyvinylidene fluorid
‐R		Relaxation
R_m_	m^−1^	Membrane resistance
*S. cerevisiae*		*Saccharomyces cerevisiae*
TMP	mbar	Transmembrane pressure
TSS		Total suspended solids
UF		Ultrafiltration
VRF	–	Volumetric reduction factor
*η*	–	Efficiency factor
*ρ*	kg/m^3^	Density

## CONFLICT OF INTERESTS

The authors have declared no conflicts of interest.

## Supporting information

Supporting information

## Data Availability

The data that support the findings of this study are available from the corresponding author upon reasonable request.
