# Supplementary material for: Characterization of an aerated submerged hollow fiber ultrafiltration device for efficient microalgae harvesting
Source: Eng Life Sci. 2021 Sep 12;21(10):607–22. doi: 10.1002/elsc.202100052 (PMC8518668; doi:10.1002/elsc.202100052)
Supplement: Supplementary file 1 — Supporting information [file ELSC-21-607-s001.pdf]

Characterization of a submerged hollow fiber ultrafiltration plant for harvesting of microalgae - Supplementary information

1. Critical flux experiments – baker's yeast and microalgae

Membrane resistance R_m before each experiment – Yeast and *Chlorella vulgaris*

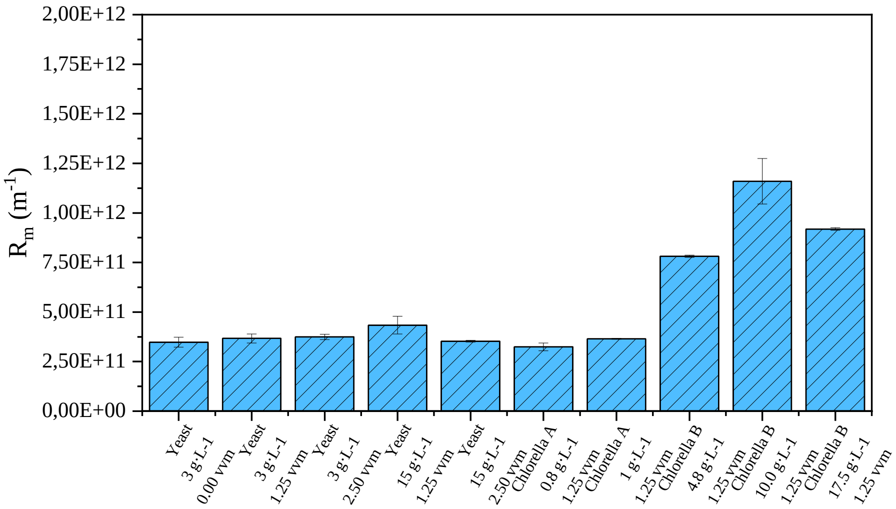

Figure S1: Membrane resistance R_m before each critical flux experiment using baker's yeast *Saccharomyces cerevisiae* and microalgae *Chlorella vulgaris* (*Chlorella* A – axenic and *Chlorella* B - non-axenic)

Filtrate Flux – transmembrane pressure (TMP) profile for critical flux experiment using non-axenic *Chlorella* B biomass of 10.0 g DW·L^{-1} and 17.5 g DW·L^{-1} (DW: dry weight)

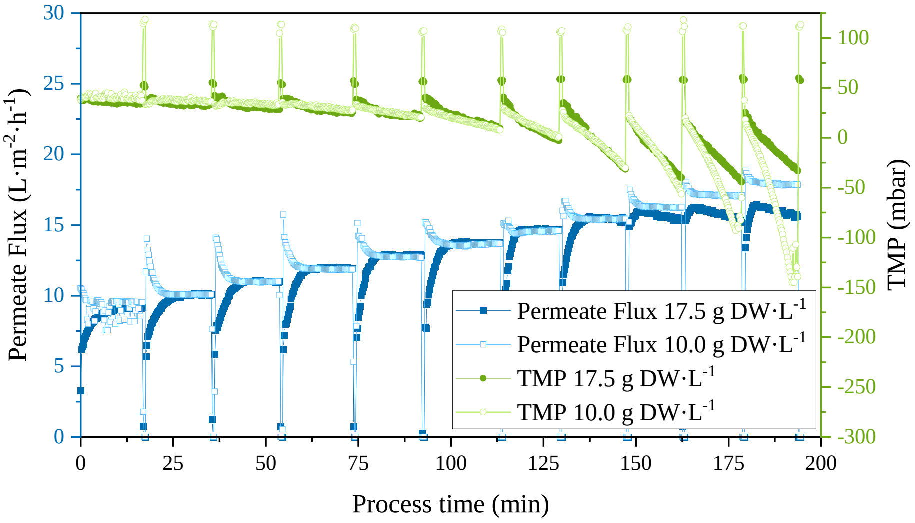

Figure S2: Filtrate Flux and transmembrane pressure (TMP) over during critical flux experiment for dry biomass (DBM) concentration of 10.0 g DW·L^{-1} and 17.5 g DW·L^{-1} (comparison)

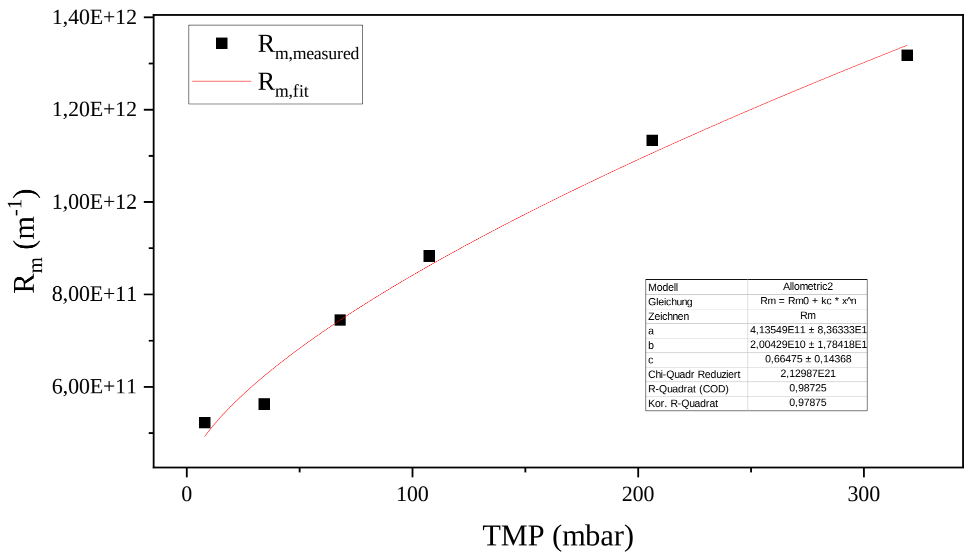

Figure S3: Increase of membrane resistance R_m with applied transmembrane pressure (TMP); measured values and allometric fit.
